# The long-term economic impacts of arthritis through lost productive life years: results from an Australian microsimulation model

**DOI:** 10.1186/s12889-018-5509-3

**Published:** 2018-05-24

**Authors:** Deborah Schofield, Michelle Cunich, Rupendra N. Shrestha, Robert Tanton, Lennert Veerman, Simon Kelly, Megan E. Passey

**Affiliations:** 1 GenIMPACT: Centre for Economic Impacts of Genomic Medicine, Department of Economics, Faculty of Business and Economics, Macquarie University, Sydney, NSW 2107 Australia; 2 The Boden Institute of Obesity, Nutrition, Exercise and Eating Disorders, Charles Perkins Centre, The University of Sydney, Sydney, NSW 2006 Australia; 3 National Centre for Social and Economic Modelling, University of Canberra, Canberra, ACT Australia; 4 School of Medicine, Griffith University, Gold Coast, QLD Australia; 5 University Centre for Rural Health, School of Public Health, The University of Sydney, Lismore, NSW Australia; 6 Cancer Council NSW, Woolloomooloo, NSW 2011 Australia

**Keywords:** Arthritis, Indirect costs, Income, Welfare payments, Taxation, GDP, Labour force participation, Microsimulation, Arthritis management

## Abstract

**Background:**

While the direct (medical) costs of arthritis are regularly reported in cost of illness studies, the 'true' cost to indivdiuals and goverment requires the calculation of the indirect costs as well including lost productivity due to ill-health.

**Methods:**

Respondents aged 45-64 in the ABS Survey of Disability, Ageing and Carers 2003, 2009 formed the base population. We projected the indirect costs of arthritis using *Health&WealthMOD2030* – Australia’s first microsimulation model on the long-term impacts of ill-health in older workers – which incorporated outputs from established microsimulation models (STINMOD and APPSIM), population and labour force projections from Treasury, and chronic conditions trends for Australia. All costs of arthritis were expressed in real 2013 Australian dollars, adjusted for inflation over time.

**Results:**

We estimated there are 54,000 people aged 45-64 with lost PLYs due to arthritis in 2015, increasing to 61,000 in 2030 (13% increase). In 2015, people with lost PLYs are estimated to receive AU$706.12 less in total income and AU$311.67 more in welfare payments per week than full-time workers without arthritis, and pay no income tax on average. National costs include an estimated loss of AU$1.5 billion in annual income in 2015, increasing to AU$2.4 billion in 2030 (59% increase). Lost annual taxation revenue was projected to increase from AU$0.4 billion in 2015 to $0.5 billion in 2030 (56% increase). We projected a loss in GDP of AU$6.2 billion in 2015, increasing to AU$8.2 billion in 2030.

**Conclusions:**

Significant costs of arthritis through lost PLYs are incurred by individuals and government. The effectiveness of arthritis interventions should be judged not only on healthcare use but quality of life and economic wellbeing.

## Background

The latest Global Burden of Disease Study (2015) estimated there were 538 million Years Lived with Disability (YLDs) globally due to acute and chronic diseases and injuries in 1990, which increased to 764.8 million in 2013 due to population growth and ageing (42% increase) [1]. Musculoskeletal disorders were a major category of chronic disease contributing to the increase in YLDs. YLDs for rheumatoid arthritis (RA) increased by 56.8% and for osteoarthritis (OA) by 75.4% between 1990 and 2013. While YLDs and other measures of disease burden are useful for evaluating and setting health policy, the indirect costs of chronic diseases are also important, and not only for health policy but related policy areas e.g. employment, finance and social security. Together, these measures provide vital information for modern governments in their pursuit of cross-portfolio solutions to complex health and social issues [2–4].

Current lost labour force participation due to arthritis – where ‘arthritis’ refers to a number of different conditions leading to inflamed or damaged joints, with the main conditions for people aged 45-64 years being OA and RA [5] – will impact on the future capacity of patients to maintain an adequate standard of living [6] and future governments to have sufficient revenue from which to fund the healthcare needed by the ageing population [2, 3]. Many governments are seeking new ways to make efficiency gains because the pool of workers is diminishing due to low fertility rates and population ageing [2]. Thus there is an urgency to calculate the indirect costs of chronic diseases for individuals and the government. It is widely acknowledged that investing in health contributes to the objectives of “smart, sustainable and inclusive growth” [4].

The direct costs of arthritis are substantial and rising. The main driver for these costs is the increasing prevalence of arthritis with ageing. In Australia, there are 3.9 million people with arthritis and this population is projected to grow to 5.4 million by 2030. Arthritis currently costs the health system $5.5 billion and these costs are projected to grow to $7.6 billion by 2030 [7].

However the indirect costs of arthritis are considered to be greater than the direct costs [7–9]. These extra costs are mostly due to lost productivity, with arthritis affecting an individual’s ability to maintain employment due to pain and physical disability [8, 10]; but also costs associated with the need for informal carers [11]. A recent study has shown that of the Australians aged 45-64 years who are out of the labour force due to ill-health, 13.3% were out because of arthritis in 2010 (45,000 people) [12]. Consequently, arthritis is the second most common chronic condition (after back problems) causing people to leave the labour force among those aged 45-64 years [12].

Commonwealth Treasury’s *Intergenerational Report* (IGR) 2015 [2] highlights that population ageing and labour shortages are the main challenges facing the government in terms of budget sustainability. In response to these challenges, the Australian Government has sought to increase the Age Pension eligibility to 70 years by 2035 and implement other policies to encourage deferral of retirement. From 2012 to 2061, the proportion of the working-age group (15-64 years) who are aged 50-64 is projected to increase to between 27 and 30% [13]. However this is also the age group from which 21% of men and 121% of women retire early due to own ill-health [14]. Thus the prevention and treatment of chronic conditions are crucial to keeping older workers in the labour force [15]. Importantly, a number of randomised controlled trials for the treatment of arthritis have demonstrated effectiveness in relation to increased labour force participation [8, 16]. Additionally, adjustments to relevant work-related factors can reduce the risk of work disability in people with arthritis (such as self-employment, modification of workstations, family support for the person with arthritis maintaining employment, reducing commuting difficulty, and increasing comfort for telling colleagues about arthritis) and thereby increase their employment status/duration. The aim of this study was to project the indirect costs of arthritis due to lost productive life years (PLYs) – defined as *the number of people not in the labour force who would have been in the labour force were it not for their arthritis in a given year* [12] – from 2015 to 2030 using a microsimulation model. We note that there has been a recent change in the United States’ recommendations on cost-effectiveness analysis which highlights a move from excluding productivity costs to now including them [17, 18]. This change suggests a specific need for the type of cost data that the current study presents.

## Methods

**Fig. 1:**
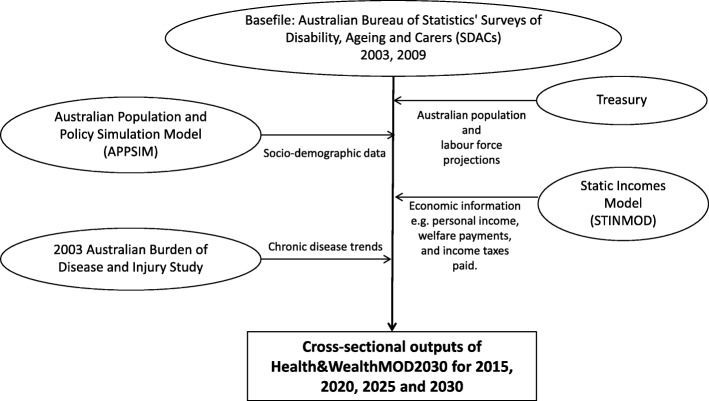
Diagram of the Health&WealthMOD2030 model

A microsimulation model, Health&WealthMOD2030, was used to project the indirect costs of arthritis for individuals and the government from 2015 to 2030. The data sources and statistical methods used to develop Health&WealthMOD2030 are discussed in [19]. Figure 1 provides a graphical representation of the microsimulation model, Health&WealthMOD2030, used in this study.

### Base population

The base population of Health&WealthMOD2030 consists of unit-record data (people aged 45-64) from the ABS *Survey of Disability, Ageing and Carers* (SDACs) *2003* and *2009* [20] – nationally representative household surveys. Personal (age, sex and family type), socioeconomic (education, labour force participation, income, receipt of welfare payments and type of payments) and health characteristics (main chronic condition) for each individual in the household were extracted.

### Economic data

The National Centre for Social and Economic Modelling’s (NATSEM, University of Canberra) Static Incomes Model or STINMOD is the foremost microsimulation model of Australia’s income tax and cash transfer (welfare) system [21]. Income, welfare, tax and wealth information from STINMOD’s 2013 snapshot were imputed onto the base population of Health&WealthMOD2030 by identifying individuals with similar characteristics on STINMOD and “donating” their economic information onto Health&WealthMOD2030 using synthetic matching. Ten variables were used for synthetic matching: labour force status, income unit type, income quintile, receipt of Aged Pension, receipt of Disability Support Pension (DSP), sex, age group, hours of work per week, education and home ownership.

The economic data from STINMOD were indexed to match economic growth from 2013 to the projection years (2015, 2020, 2025 and 2030). Income and taxes were assumed to grow at a rate of 1% per annum in real terms and welfare payments to have no real growth – consistent with the Australian Government’s policy of only increasing welfare payments in line with Consumer Price Index (CPI) growth.

### Population, labour force and chronic disease trends

We used Commonwealth Treasury’s population and work force projections from 2015 to 2030 (by five-year age group) in our model. We applied the projected age-sex specific distributions of other socio-demographic variables (education, income unit, home ownership and receipt of Disability Support Payments, DSP) from a second NATSEM microsimulation model, the Australian Population and Policy Simulation Model (APPSIM) [22], in our modelling.

The chronic disease trends used in the model were the same as the trends in chronic condition incidence used in Begg et al.’s (2008) *2003 Australian Burden of Disease and Injury Study* [23].

### Reweighting

The SDACs 2003 and 2009 were reweighted separately using the ABS reweighting algorithm GREGWT [24] so as to account for demographic, labour force, disease prevalence and other changes between the survey years (2003 and 2009) and the projection years.

Use of SDACs 2003 and 2009 were approved by the ABS Microdata Review Panel.

### Lost productive life years (PLYs) due to arthritis

All SDAC respondents who indicated (a) they were not in the labour force due to their ‘own ill-health or disability’, and (b) nominated their main health condition to be “arthritis and related disorders” (ICD10 code M00-19) were considered to have lost PLYs due to arthritis. Severity of arthritis is not collected in the SDACs although it is likely that those who are out of the labour force due to their condition have more severe arthritis.

### Indirect costs

The indirect costs of arthritis through lost PLYs consisted of lost income, extra welfare payments, and lost taxation revenue. Personal income consisted of earnings, income from other sources producing a return, and welfare payments. Relevant welfare payments were: Disability Support Pension (DSP), Newstart Allowance, Carer Payment, and Family Tax Benefit (http://www.humanservices.gov.au). We note that the incremental welfare costs associated with reduced labour force participation for people with arthritis (versus those without) are most likely the costs of the DSP. The taxes paid by individuals included personal income tax and the Medicare levy.

We calculated the impact of arthritis on GDP in each year using projections and methods for estimating GDP and the impact of the work force from the Commonwealth Treasury [25].

### Statistical analysis

We present a summary of the number of people with and without lost PLYs due to arthritis, and the mean (standard deviation) and median income, welfare payments and taxes paid per week by people aged 45-64 years in 2015, 2020, 2025 and 2030. All figures are expressed in real 2013 Australian dollars (AU$) (that is, adjusted for changes due to inflation [CPI] over time).

A quantile regression model for median weekly income with age, sex and education as explanatory variables was used to estimate the difference between the income of people with lost PLYs due to arthritis versus full-time workers without arthritis in 2015, 2020, 2025 and 2030. Similar models for median weekly welfare payments and median weekly taxes were also estimated.

The national costs of arthritis through people aged 45-64 years exiting the labour force were projected from 2015 to 2030. The total income loss at a national level were estimated based on the differences between the income of those with lost PLYs due to arthritis and the income of those in full-time work with no arthritis and the income of those in part-time work with no arthritis and their probabilities of being in full-time, part-time work or unemployed if they did not have arthritis. 95% Confidence Intervals (CIs) were generated for national income, welfare payments and taxes using bootstrapping with 1000 replications for each year.

The statistical analysis was conducted in SAS V9.4 (SAS Institute Inc., Cary, NC, USA), and all statistical tests were two-sided with a 5% level of significance.

## Results

Among the 5,712,000 people aged 45-64 years in 2015, it was estimated that 54,000 (0.9%) were out of the labour force due to arthritis; 307,000 (5.2%) were working full-time with arthritis; 186,000 (3.1%) were working part-time with arthritis; 2,912,000 (49%) were working full-time without arthritis; and 1,032000 (17.4%) were working part-time without arthritis (Table 1).

**Table 1:** Mean and median weekly income, welfare payments and taxes of individuals with and without arthritis as main chronic condition, Australian population aged 45-64 years (in real 2013 Australian dollars) (crude estimates)

	N	2015				2020				2025				2030			
Labour force status^a^	Survey records	Weighted population (%)	Mean	SD	Median	Weighted population (%)	Mean	SD	Median	Weighted population (%)	Mean	SD	Median	Weighted population (%)	Mean	SD	Median
Weekly total income (AU$) of individuals^b^																	
Working full-time without arthritis	11,562	2,912,000 (48.98)	1597.29	1558.02	1308.88	3,169,000 (49.72)	1721.67	1675.99	1427.46	3,336,000 (49.96)	1864.91	1831.87	1521.93	3,600,000 (50.49)	2000.81	1969.95	1610.67
Employed full-time with arthritis	1120	307,000 (5.16)	1366.59	1116.91	1180.65	339,000 (5.32)	1473.12	1193.94	1253.34	358,000 (5.37)	1632.74	1261.08	1378.67	379,000 (5.31)	1769.93	1345.96	1468.07
Employed part-time without arthritis	4477	1,032000 (17.36)	722.14	794.20	602.86	1,154,000 (18.11)	773.80	853.10	640.03	1,234,000 (18.94)	853.12	892.45	700.84	1,340,000 (18.79)	925.08	921.38	767.07
Employed part-time with arthritis	708	186,000 (3.13)	597.41	551.25	551.77	209,000 (3.27)	636.79	594.11	582.75	222,000 (3.33)	729.29	655.05	665.73	236,000 (3.31)	821.04	700.99	741.10
Not in labour force due to arthritis	208	54,000 (0.91)	334.06	148.69	321.87	58,000 (0.91)	336.85	152.90	330.33	59,000 (0.88)	338.72	158.50	340.91	61,000 (0.85)	340.60	167.15	352.05
Weekly welfare income (AU$) received by individuals^c^																	
Employed full-time without arthritis	11,562	2,912,000 (48.98)	18.27	62.34	0.00	3,169,000 (49.72)	17.66	61.56	0.00	3,336,000 (49.96)	15.76	58.18	0.00	3,600,000 (50.49)	14.91	56.80	0.00
Employed full-time with arthritis	1120	307,000 (5.16)	19.92	71.13	0.00	339,000 (5.32)	20.58	73.24	0.00	358,000 (5.37)	19.58	72.40	0.00	379,000 (5.31)	18.81	71.38	0.00
Employed part-time without arthritis	4477	1,032000 (17.36)	73.41	140.41	0.00	1,154,000 (18.11)	72.51	140.40	0.00	1,234,000 (18.94)	66.73	134.93	0.00	1,340,000 (18.79)	63.04	130.49	0.00
Employed part-time with arthritis	708	186,000 (3.13)	100.13	162.92	0.00	209,000 (3.27)	99.14	164.29	0.00	222,000 (3.33)	89.86	157.81	0.00	236,000 (3.31)	84.67	153.38	0.00
Not in labour force due to arthritis	208	54,000 (0.91)	305.51	158.51	311.67	58,000 (0.91)	303.89	158.00	311.67	59,000 (0.88)	298.84	161.70	311.67	61,000 (0.85)	294.15	163.83	311.67
Weekly tax paid (includes Medicare levy) (AU$) by individuals^d^																	
Employed full-time without arthritis	11,562	2,912,000 (48.98)	353.62	488.20	243.18	3,169,000 (49.72)	385.42	530.06	264.97	3,336,000 (49.96)	420.48	576.40	291.01	3,600,000 (50.49)	454.06	624.27	310.31
Employed full-time with arthritis	1120	307,000 (5.16)	278.53	358.89	184.99	339,000 (5.32)	303.50	389.78	198.14	358,000 (5.37)	340.43	421.35	226.16	379,000 (5.31)	372.05	457.59	252.38
Employed part-time without arthritis	4477	1,032000 (17.36)	84.29	203.91	19.08	1,154,000 (18.11)	93.27	220.83	20.79	1,234,000 (18.94)	104.28	229.82	29.74	1,340,000 (18.79)	113.01	236.16	37.87
Employed part-time with arthritis	708	186,000 (3.13)	49.73	118.28	0.00	209,000 (3.27)	55.06	130.88	0.00	222,000 (3.33)	67.36	147.57	7.63	236,000 (3.31)	78.92	163.70	24.59
Not in labour force due to arthritis	208	54,000 (0.91)	−0.16	6.79	0.00	58,000 (0.91)	0.04	9.50	0.00	59,000 (0.88)	0.03	9.89	0.00	61,000 (0.85)	0.07	11.37	0.00

^a^There were 25,104 people aged 45-64 years in the concatenated SDAC 2003 and 2009 data. Of these, 18,075 people were identified as being in one of the labour force categories listed in Table 1. A further 5827 were unemployed or not in the labour force due to reasons other than ill-health, and 1202 were not in the labour force due to ill-health with conditions other than arthritis as the main condition. The total number of survey records was 24,253. The weighted population was 5,945,000 in 2015; 6,374,000 in 2020; 6,677,000 in 2025; and 7,130,000 in 2030

^b^Personal income consisted of labour market earnings, income from other sources generating a return (such as rental properties, investments, interest on cash in a bank), and welfare payments

^c^Relevant welfare payments for this age group consisted of disability support pension, Newstart Allowance (for people looking for work), Carer Payment, and Family Tax Benefit; see https://humanservices.gov.au

^d^The taxes paid by individuals included personal income tax and the Medicare levy

Those who were out of the labour force due to arthritis received an estimated $321.87 in median income per week in 2015, which is only a quarter of the median income of full-time workers without arthritis (AU$1308.88 per week) (Table 1). Those not in the labour force due to arthritis also received median welfare payments of AU$311.67 per week (Table 1).

By 2030, the older working-age population was projected to be 6,843,000 and consisted of 61,000 people with lost PLYs due to arthritis, 379,000 working full-time with arthritis, 236,000 working part-time with arthritis; 3,600,000 working full-time without arthritis, and 1,340,000 working part-time without arthritis. Those with lost PLYs due to arthritis were projected to receive AU$352.05 per week in median income and AU$311.67 per week in median welfare payments in 2030 (Table 1, last column).

Compared to those in full-time employment without arthritis (adjusted for age, sex and education), people out of the labour force due to arthritis were estimated to receive AU$706.12 (95%CI: AU$606.23- AU$743.17) less per week in median income in 2015 (Table 2). They also received significantly more in welfare payments (an extra AU$311.67 per week, 95%CI: AU$310.99- AU$413.50) and pay less in taxes (AU$171.20 per week, 95% CI: AU$150.77- AU$188.20) compared to full-time workers without arthritis. The differences in median weekly income, welfare payments and taxation between those with lost PLYs due to arthritis and full-time workers without arthritis were also estimated for 2030 (Table 2, last two columns). Lost income as a result of being out of the labour force due to arthritis was projected to increase from AU$706.12 per week in 2015 (95%CI: AU$606.23- AU$743.17) to AU$970.96 per week in 2030 (95% CI: AU$920.52- AU$1034.92) in real terms (compared to those working full-time without arthritis). People with lost PLYs due to arthritis paid an estimated AU$171.20 per week (95% CI: AU$150.77- AU$188.20) less in income taxes than those working full-time without arthritis in 2015, increasing to AU$234.79 per week (95% CI: AU$205.48- AU$243.53) in 2030.

**Table 2:** Differences in median weekly income, welfare payments and taxes between older workers with lost PLYs due to arthritis and those employed full time without arthritis (adjusted for age, sex and education), Australian population aged 45-64 years (in real 2013 Australian dollars)

	2015		2020		2025		2030	
Labour force status	$ difference	95% CI	$ difference	95% CI	$ difference	95% CI	$ difference	95% CI
Weekly total income (AU$) of individuals								
Employed full-time without arthritis	*Reference group*							
Employed full-time with arthritis	−25.18	(−76.50,31.50)	−24.80	(−76.67,41.52)	−18.43	(−83.35,33.48)	− 27.61	(− 91.66,36.61)
Employed part-time without arthritis	− 565.61*	(−597.42,-534.83)	− 609.04*	(− 645.23,-571.85)	− 652.58*	(− 687.79,-619.42)	− 698.11*	(− 726.50,-658.14)
Employed part-time with arthritis	− 578.81*	(− 642.87,-521.67)	− 635.00*	(− 693.26,-553.52)	−652.09*	(− 738.18,-567.82)	− 684.46*	(− 744.36,-617.03)
Not in labour force due to arthritis	− 706.12*	(−743.17,-606.23)	− 762.30*	(− 808.81,-664.20)	− 873.26*	(− 923.61,-815.25)	−970.96*	(−1034.92,-920.52)
Weekly welfare income (AU$) received by individuals								
Employed full-time without arthritis	*Reference group*							
Employed full-time with arthritis	0.00	(0,0)	0.00	(0,0)	0.00	(0,0)	0.00	(0,0)
Employed part-time without arthritis	0.00	(0,0)	0.00	(0,0)	0.00	(0,0)	0.00	(0,0)
Employed part-time with arthritis	0.00	(0,5.75)	0.00	(0,5.75)	0.00	(0,0)	0.00	(0,0)
Not in labour force due to arthritis	311.67*	(310.99,413.50)	311.67*	(301.14,413.50)	311.67*	(311.67,413.50)	311.67*	(311.67,396.16)
Weekly tax paid (includes Medicare levy) (AU$) by individuals								
Employed full-time without arthritis	*Reference group*							
Employed full-time with arthritis	−26.31*	(−44.46,-3.75)	− 22.73*	(− 46.68,-3.02)	−23.56	(−40.13,1.91)	−17.17	(−38.82,5.48)
Employed part-time without arthritis	−158.54*	(− 166.71,-148.13)	−172.76*	(− 182.94,-161.71)	− 195.21*	(− 207.55,-185.22)	−208.60*	(− 221.35,-198.15)
Employed part-time with arthritis	− 158.54*	(− 165.99,-145.28)	− 172.76*	(−182.76,-157.18)	− 184.50*	(−202.18,-163.50)	−193.13*	(− 215.50,-177.55)
Not in labour force due to arthritis	−171.20*	(−188.20,-150.77)	− 180.64*	(−200.50,-164.44)	−210.77*	(−221.79,-190.51)	−234.80*	(−243.53,-205.48)

*p-value < 0.05

**Table 3:** National costs of lost workers (full- and part-time) due to arthritis per year (in real 2013 Australian dollars, millions)

	2015		2020		2025		2030	
Cost	$ impact	95% CI	$ impact	95% CI	$ impact	95% CI	$ impact	95% CI
Lost income	1516	(1222; 1725)	1756	(1429; 2019)	2086	(1739; 2433)	2406	(2012; 2826)
Extra welfare payments	847	(732; 1162)	912	(782; 1244)	933	(548; 1261)	959	(823; 990)
Lost tax revenue	352	(279; 420)	394	(321; 476)	473	(383; 548)	549	(422; 615)

Lost workers per year:

Of the 54,000 people out of the labour force due to arthritis in 2015, it is projected 39,000 (71.68%) move into full-time employment and 14,000 (25.40%) move into part-time employment (the residual 1000 remain in unemployment)

Of the 58,000 people out of the labour force due to arthritis in 2020, it is projected 41,000 (71.46%) move into full-time employment and 15,000 (26.02%) move into part-time employment (the residual 2000 remain in unemployment)

Of the 59,000 people out of the labour force due to arthritis in 2025, it is projected 42,000 (71.24%) move into full-time employment and 16,000 (26.37%) move into part-time employment (the residual 1000 remain in unemployment)

Of the 61,000 people out of the labour force due to arthritis in 2030, it is projected 43,000 (71.17%) move into full-time employment and 16,000 (26.49%) move into part-time employment (the residual 2000 remain in unemployment)

**Table 4:** Lost GDP owing to missing workers aged 45-64 years due to arthritis, 2015-2030 (in real 2013 Australian dollars, millions)^a^

	2015	2020	2025	2030
Projected GDP	$1,483,861	$1,678,852	$1,899,467	$2,149,073
Lost GDP owing to missing workers due to arthritis*	$6208	$6852	$7535	$8191
Potential % gain in total GDP if able to keep missing workers due to arthritis in the labour force	0.42	0.41	0.40	0.38

^a^Impacts are based on projections of 53,000, 56,000, 58,000 and 59,000 missing workers (full-time and part-time) due to arthritis in 2015, 2020, 2025 and 2030, respectively

The national impact of arthritis through lost PLYs consists of an estimated $1516 million (95%CI: AU$1222 million- AU$1725 million) in lost income in 2015, increasing to AU$2406 million (95%CI: AU$2012 million- AU$2826 million) in 2030 (i.e. a 59% increase in lost income for this period) mainly due to ageing. Additional welfare payments paid as a result of people leaving the labour force because of arthritis were projected to increase by 13% over the period, from AU$847 million (95%CI: AU$732 million to AU$1162 million) in 2015 to $959 million (95%CI: $ AU823 million- AU$990 million) in 2030 (Table 3). Finally, lost annual taxation revenue was projected to increase by 56% in real terms, from AU$352 million (95%CI: AU$279 million- AU$420 million) in 2015 to AU$549 million (95%CI: AU$422 million- AU$615 million) in 2030.

We calculated the GDP losses attributable to older workers leaving the labour force because of arthritis to reach AU$6.2 billion, AU$6.8 billion, AU$7.5 billion, and AU$8.2 billion in 2015, 2020, 2025 and 2030, respectively (Table 4). If those people were able to undertake arthritis management that kept them in the work force then we predict a potential gain in GDP of 0.4% per year.

## Discussion

The study projected that 54,000 people aged 45-64 years have lost PLYs due to arthritis in 2015, increasing to 61,000 in 2030 – a 13% increase. The national impacts of lost PLYs due to arthritis consisted of a 59% increase in lost income, which grew faster than the 13% increase in welfare payments due to the indexation of welfare payments being less than expected wages growth; and a 32% increase in lost GDP. The projection of long-term costs of arthritis, and the calculation of welfare payments and lost taxes are new contributions to the literature. These costs are in addition to the substantial health (direct) costs of arthritis. It is estimated that in the USA, $185.5 billion in annual insurer expenditures are attributable to medical care for patients with OA [26].

There are some limitations to the study. One is that the study findings are based on SDAC respondents self-reporting work status and chronic diseases; although self-reported work status and health are considered to be valid measures for costing studies [27, 28]. Another is that the study focuses on measuring productivity as labour force participation and does not consider other forms such as presenteeism and absenteeism.

The main benefit of using large-scale microsimulation models (such as Health&WealthMOD2030) is that they are based on micro data from national surveys conducted by the Australian Bureau of Statistics and therefore can be used to examine the impact of social policy changes on particular subgroups or demographics [29]. The models emulate the heterogeneity in the population due to the base population (and trends) being derived from a large household survey. Other benefits are that microsimulation models can be developed in such a way as to reproduce the complexity of policy arrangements, transfers and settings and consequently can be used to predict the outcomes of changes in policy using “what if” scenarios [30]. Thus Health&WealthMOD2030 can be used to calculate the impacts of effective interventions to prevent or delay chronic diseases (such as non-surgical management of knee OA [8]) on potential cost savings for individuals and the government.

A number of randomised controlled trials for arthritis treatment have demonstrated effectiveness in terms of increased labour force participation [31]. Additionally, Lacaille et al. (2004) found that modifying work-related factors that increase the risk of work disability in people with arthritis (such as options for self-employment, workstation modification, work importance, family support towards employment, commuting difficulty, and comfort telling colleagues about arthritis) can increase employment [32]. In Australia, self-managed interventions to overcome workplace challenges associated with chronic pain have shown effectiveness. These results suggest that the government could achieve cost savings if effective arthritis management in workers occurred prior to their ill-health dictating withdrawal from the labour force, provided the costs to prevent arthritis outweigh the losses in indirect costs. In Australia, there are RCTs of self-management interventions to help overcome workplace challenges associated with chronic pain (such as musculoskeletal pain and arthritis) which have demonstrated effectiveness; for example, the ‘ADAPT’ for work-related pain program [33]. ADAPT is an intensive cognitive-behavioural pain management program, run by the Pain Management and Research Centre at the University of Sydney and Royal North Shore Hospital (http://sydney.edu.au/medicine/pmri/patient-services/resources/index.php), with a key focus on assisting people to better manage their pain and, in turn, their engagement with the work place [34]. The current study involved modelling the impact of arthritis on PLYs within the current treatment regimes. Future work could assess the likely impact of specific interventions that change the status quo and/or business as usual treatment for arthritis.

Quality of life is the most common measure of the benefits (or health outcomes) for patients in cost-effectiveness studies of health interventions. However, the results of the present study suggest that, not only would the amount of healthcare resources consumed by patients (direct medical costs) be important components of cost-effectiveness studies but also the indirect costs such as changes in labour market participation due to illness. The focus of this paper has been on quantifying the magnitude of the indirect costs of arthritis through lost labour force participation. We would also maintain that these additional costs are important considerations for policymakers in determining appropriate allocations of resources in health, social security, employment and other portfolios. The sentiments expressed in this paper are consistent with the recent change in the United States’ recommendations on cost-effectiveness analysis which emphasises a move away from excluding (or ignoring) productivity costs to now including them in analysis [17, 18]. Thus there is a particular need for the type of cost inputs that the current study presented.

## Conclusions

Until recently, public policy has focused on economic incentives to increase labour force participation. However, as chronic disease is a key barrier to labour force participation in older workers, with arthritis being one of the diseases having the greatest impact on work capacity, more investment in health interventions to address chronic diseases are also needed.
